# Emerging Roles of Xanthine Oxidoreductase in Chronic Kidney Disease

**DOI:** 10.3390/antiox13060712

**Published:** 2024-06-12

**Authors:** Hunter W. Korsmo, Ubong S. Ekperikpe, Ilse S. Daehn

**Affiliations:** Department of Medicine, Division of Nephrology, The Icahn School of Medicine at Mount Sinai, One Gustave Levy Place, Box 1243, New York, NY 10029, USA

**Keywords:** XOR, reactive oxygen species, endothelial injury, kidney disease, diet

## Abstract

Xanthine Oxidoreductase (XOR) is a ubiquitous, essential enzyme responsible for the terminal steps of purine catabolism, ultimately producing uric acid that is eliminated by the kidneys. XOR is also a physiological source of superoxide ion, hydrogen peroxide, and nitric oxide, which can function as second messengers in the activation of various physiological pathways, as well as contribute to the development and the progression of chronic conditions including kidney diseases, which are increasing in prevalence worldwide. XOR activity can promote oxidative distress, endothelial dysfunction, and inflammation through the biological effects of reactive oxygen species; nitric oxide and uric acid are the major products of XOR activity. However, the complex relationship of these reactions in disease settings has long been debated, and the environmental influences and genetics remain largely unknown. In this review, we give an overview of the biochemistry, biology, environmental, and current clinical impact of XOR in the kidney. Finally, we highlight recent genetic studies linking *XOR* and risk for kidney disease, igniting enthusiasm for future biomarker development and novel therapeutic approaches targeting XOR.

## 1. Introduction

Xanthine oxidoreductase (XOR) is the rate-limiting enzyme in the terminal steps of purine metabolism, which is the process of generating the building blocks of DNA and RNA. XOR’s reaction results in the production of uric acid (UA) in humans, of which two-thirds are excreted by the kidneys into urine, while the remaining one-third is eliminated via the gut. XOR can also produce reactive oxygen species (ROS) as it oxidizes hypoxanthine to xanthine as well as xanthine to UA [1,2,3]. XOR activity can be influenced by environmental factors, such as purine-rich and Western diets, infections, and increasing temperatures. Various disease states such as diabetes and aging can also enhance XOR activity [4,5]. Consequently, hyperuricemia (defined as elevated levels of serum UA) together with the generation of excess ROS can elicit damage to tissues, particularly on the endothelium [6,7,8,9,10].

XOR-derived ROS provides protection against infection, however, excess ROS through this pathway can also lead to inflammation and the development of oxidative distress-related tissue injury [11]. XOR-derived UA is considered a causal metabolite in the development of gout and a risk factor in the development of chronic kidney disease (CKD) and cardiovascular disease [12,13,14,15,16]. The presence of XOR in the blood can be used as a biomarker for a number of conditions, hence, XOR has been a drug target of interest for nearly 70 years and XOR inhibitors, either purine mimetics or selective non-purine drugs, have been effective at lowering serum UA levels [11,15,17,18,19,20,21,22]. However, these drugs have many limitations to their long-term use. Recent studies have found a correlation between genetic variants that regulate XOR activity and susceptibility to organ damage, suggesting a significant role of *XOR* and its products in this process [5,23]. In this review, we summarize the biochemical, physiological, and clinical implications of enhanced XOR activity and highlight the mechanistic implications of endothelial activation and ROS interplay in CKD.

## 2. XOR Biochemistry

**Figure 1:**
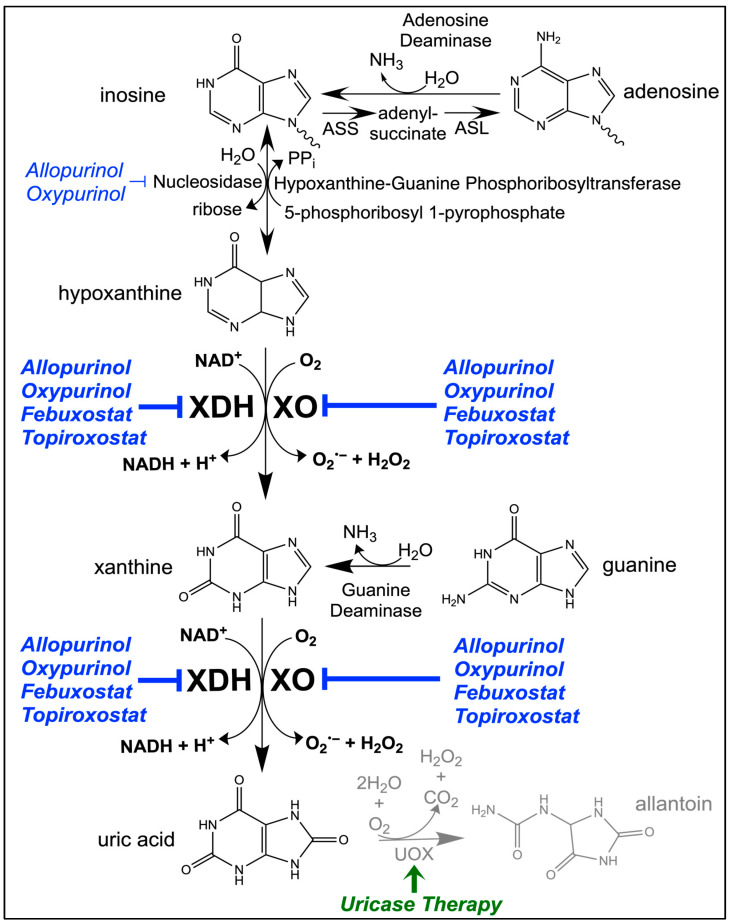
**Terminal Steps of Purine Catabolism Mediated by XOR.** Purines are predominantly metabolized to hypoxanthine, which is subsequently oxidized to xanthine and further to UA. Inhibitors at various steps are indicated (Blue), and purine mimetics have off-target effects (Blue, thin print). The pseudogenized human UOX previously produced allantoin (Grey). Uricase therapy (Green) can promote this pathway in humans. XOR, xanthine oxidoreductase; XDH, xanthine dehydrogenase; XO, xanthine oxidase; UA, uric acid; ASL, adenine succinate lyase; ASS, adenine succinate synthase; NAD(H), nicotinamide adenine dinucleotide; PP_i_, inorganic pyrophosphate; UOX, urate oxidase.

XOR catalyzes the oxidation of hypoxanthine to xanthine and xanthine to UA whilst concomitantly reducing NAD^+^ to NADH as a dehydrogenase (xanthine dehydrogenase; XDH) or generates ROS, superoxide (O_2_^•−^) and hydrogen peroxide (H_2_O_2_) in its oxidase (xanthine oxidase; XO) form [10,11]. XOR is a molybdo-flavoenzyme and exists as a homodimer, with each subunit comprising an essential molybdenum cofactor (MoCo), two iron-sulfur clusters ([2Fe-2S] centers), and a flavin adenine dinucleotide (FAD) moiety where NAD^+^ binds [24]. The MoCo center facilitates substrate-level oxidation via water, and the subsequent electron transfer through the [2Fe-2S] centers to FAD, which in the case of XO, transfers the electrons to molecular oxygen producing O_2_^•−^ and H_2_O_2_ [25,26]. Cofactor availability also can impact the activity of XOR, particularly by the presence of NAD^+^, which competes with molecular oxygen at the FAD site of XDH [25]. XDH oxidizes NAD^+^ and generates H^+^ from water forming NADH [Figure 1, below]. XDH requires ~500µM NAD^+^ to generate uric acid at its maximal activity, whereas XO produces UA independent of physiological NAD^+^ levels, underscoring the competitiveness of the FAD site between NAD^+^ (*K_d_* = 25 µM) and O_2_ [25,27]. Moreover, studies have demonstrated the conserved local sulfhydryl groups of XDH, Cys^535^, Cys^992^, Cys^1316^, and Cys^1324^ play an effective role in regulating the post-translational conversion of XDH to XO, ultimately determining the sensitivity of the FAD site for O_2_ binding and subsequent generation of ROS [28]. This conversion from XDH to XO through oxidation is reversible; however, under prolonged ischemic conditions and diabetes, the conversion occurs irreversibly via proteolysis [29,30]. UA is the endpoint of purine metabolism in humans and some primates, while allantoin is the endpoint in most mammals [31]. UA is oxidized by uricase (UOX) to form allantoin. Allantoin results from a ring-opening oxidation of the purine skeleton at the C-2 position, which improves water solubility. Animals with UOX improve renal elimination of products of purine catabolism compared to humans who lack UOX which can accumulate pathological levels of UA [32].

UOX was once responsible for further oxidizing UA in early hominids, producing a much more water-soluble (i.e., readily dissolves in urine for excretion relative to UA) allantoin [Figure 1, below]. UOX has been pseudogenized in humans and was previously active in the human peroxisome where XOR has been documented to subcellularly localize, like it does in rat and taurus livers [31,33,34]. Generation of H_2_O_2_, under these circumstances, would have restricted effusion from the peroxisome and maintained the redox state through catalase activity, the enzyme responsible for decomposing H_2_O_2_ to water and O_2_ [35,36]. In humans the kidney plays a critical role in the regulation of serum UA levels through the reabsorption of filtered UA, as well as the secretion of urate via urate transporters [37]. Thus, without UOX, XOR production of UA must be well-regulated to prevent the pathological accumulation of UA.

## 3. XOR Biology: Products and Functions

Surplus or deficiency in XOR can result in increased or decreased serum UA levels (hyperuricemia or hypouricemia, respectively) and are associated with several prevalent diseases including hypertension, CKD, and hereditary xanthinuria [38,39,40,41]. Xanthinuria is a rare genetic disorder causing the accumulation of xanthine in blood and urine, which can lead to xanthine urolithiasis or kidney stones and subsequent renal failure [42]. Mutations in exon 16 in *XDH* result in xanthinuria, limiting the function of XOR and therefore hindering the oxidation of xanthine, leading to the accumulation of xanthine crystals in the kidney. The low levels of UA (i.e., hypouricemia) can also lead to renal damage causing the accumulation of purines, and an imbalance of the redox state promoting oxidative distress [43,44,45]. On the other hand, hyperuricemia has also been linked to renal pathogeneses, likely through increased UA and/or through the excess generation of ROS in tissues, including endothelial damage and dysfunction by decreasing endothelial NO availability [43,44,46].

### 3.1. XOR Activity and Cell Damage

The role of XOR and its products in mediating cell damage has been previously described to occur via several mechanisms. Studies have shown that following entry into the cell, XOR exerts prooxidant effects through the increased production of reactive species such as O_2_^•−^, H_2_O_2_, UA and isoprostanes [47,48,49]. It can also activate NADPH oxidase (NOX) and endothelin-1 expression [50,51,52]. In endothelial cells, this would result in the activation and promotion of pro-inflammatory activity due to increased vascular endothelial permeability and local vasodilation. It has been also demonstrated that endothelium-bound XOR can inhibit NO-dependent cyclic guanosine monophosphate production in smooth muscle cells, therefore, contributing to impaired vasorelaxation states [53].

Mitochondria play a preeminent role in cellular metabolic processes such as oxidative phosphorylation, during which O_2_^•−^ is also produced [44]. Hyperuricemia has been shown to mediate damage in endothelial cells by activating the mitochondrial sodium-calcium exchanger (NCX_Mito_), which results in mitochondrial calcium overload and the increased production of O_2_^•−^ [54]. UA-mediated alterations in mitochondrial morphology and reduced intracellular ATP production have also been implicated in endothelial dysfunction [55]. Studies by Huang et al. have shown that one mechanism through which UA stimulates ROS production and mediates endothelial cell dysfunction is via the activation of aldose reductase [56]. Aldose reductase is an important enzyme of the polyol pathway that catalyzes the conversion of glucose to sorbitol, which is a process that generates large amounts of intracellular ROS [57]. The major consequences of UA-mediated ROS production include cellular DNA damage, protein damage, and lipid peroxidation [51,58,59], which can lead to inactivation of intracellular enzymes, activation of maladaptive responses that promote proinflammatory signaling cascades, and cell death [59,60].

**Figure 2:**
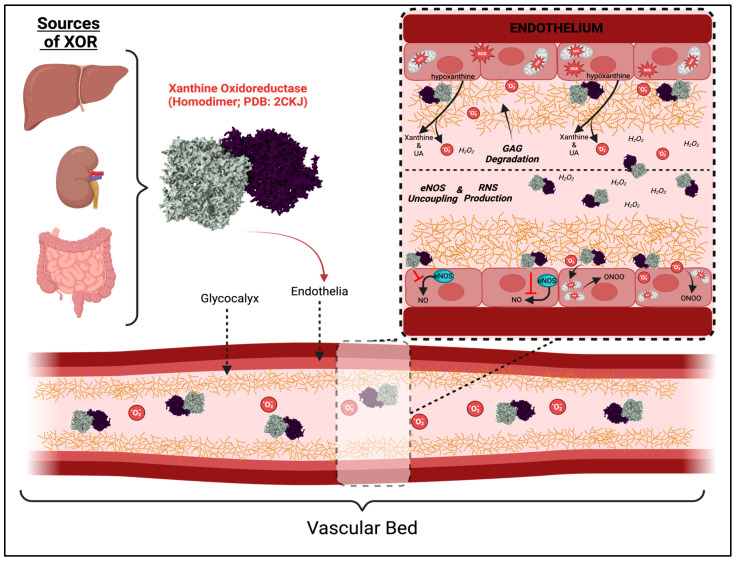
**XOR Impacts Endothelial Structure & Function.** XOR is released by multiple sources, predominantly the liver. Upon release, XOR enters the vasculature and binds to the extracellular GAG-rich glycocalyx produced by endothelial cells. Inset: XOR can contribute to the breakdown of the glycocalyx initiate endothelial activation and promote intracellular ROS production. XOR can also be endocytosed following glycocalyx binding, which may impact endothelial cell function. Additionally, XOR functions to uncouple eNOS, inhibiting NO production, and instigating ONOO- formation. eNOS, endothelial nitric oxide synthase; GAG, glycosaminoglycan; ROS, reactive oxygen species; XOR, xanthine oxidoreductase; ONOO-, peroxynitrite.

The kidney consists of a heterogeneous population of cells that carry out several energy-demanding physiological processes. Indeed, mitochondrial content and oxygen consumption of renal cells such as tubular epithelial cells (TEC) are amongst the highest in the body [61,62,63]. The implication of this is that interference with cellular metabolic processes can be pathogenic to TECs and interstitial cells, as well as cells within the glomerular compartment including podocytes, glomerular endothelial cells, and mesangial cells. UA in hyperuricemia stimulates epithelial-to-mesenchymal transition (EMT) in tubules and activates profibrotic signaling pathways and proteins like TGFβ-SMAD3 and MMPs [64,65,66]. UA has also been shown to stimulate the TLR4/NFκB signaling pathway which is involved in renal inflammation and fibrosis [64]. In the vasculature, XOR induces endothelial dysfunction via various mechanisms, including increased ROS and inhibition of endothelial nitric oxide synthase (eNOS) expression and nitric oxide (NO) release [Figure 2, below]. Additionally, UA induces endothelial-to-mesenchymal transition, mesangial cell proliferation through NOX/ROS/ERK signaling, and the increased expression of profibrotic markers in mesangial cells such as TGFβ, α-SMA, and fibronectin [67,68,69,70]. These mechanisms contribute to glomerulosclerosis and the breakdown of the glomerular filtration barrier resulting in proteinuria and progressive disease [71].

In oxidative distress states, such as those seen in diabetes, XOR activity inhibition was shown to attenuate oxidative distress and protect against diabetic kidney injury by inhibiting the vascular endothelial growth factor (VEGF)-NOX signaling pathway in human glomerular endothelial cells [72]. The study showed that increased XOR in STZ-induced diabetic kidney disease was associated with increased VEGF/VEGFR1 and VEGFR3 levels in the kidneys, followed by the activation of NOX1, NOX2, and NOX4 expression, also by FoxO3a phosphorylation and the activation of eNOS. Other studies demonstrated that XOR inhibition can prevent mitochondrial oxidative DNA damage in endothelial cells, which is characteristic of early injury [5,73]. It has also been suggested by some authors that XOR can be endocytosed by endothelial cells, thus inducing endothelial dysfunction; however, more investigations are required to validate such a mechanism [74]. In all, a potential feed-forward interplay exists between XOR-derived ROS enhancing mitochondrial-derived ROS release and endothelial cell stress [Figure 2, below], which could provide a mechanism that would promote a vicious cycle of sustained stress and accumulation of oxidized products within the microvasculature.

### 3.2. Role of XOR in CKD

Dysregulation of XOR activity has been implicated in the pathogenesis of various cardiorenal and metabolic disease states. In the context of CKD, XOR contributes to both the development and progression through direct and indirect mechanisms. Hyperuricemia due to increased XOR activity can result in the increased deposition of poorly soluble urate crystals in the renal tubules and medullary interstitial space [75]. This can lead to gouty nephropathy, which is characterized by urate kidney stones and tubular injury and can ultimately lead to renal failure [76,77]. Preclinical and clinical studies have demonstrated that hyperuricemia contributes to CKD via activation of the Nod-like receptor protein 3 (NLRP3) inflammasome-mediated inflammation and downstream pro-inflammatory events such as increased renal expression of IL1β and IL18 with increased macrophage activation and infiltration [78,79,80]. Other reports have shown that XOR-mediated renal inflammation is indeed an important pathological mechanism for the development of glomerular and tubular injury, and renal fibrosis seen in AKI and CKD possibly through the action of XOR-derived ROS [67,81,82,83,84,85,86]. Additionally, hyperuricemia can activate the intrarenal renin-angiotensin-aldosterone system (RAAS), thereby resulting in vasoconstriction of glomerular arterioles leading to glomerular ischemia, glomerular endothelial dysfunction, glomerulosclerosis, and renal fibrosis [47,87,88].

In obese diabetic mice, increased XOR activity was shown to be correlated with adipose tissue inflammation, and pharmacological blockade of XOR rescued markers of metabolic syndrome in these mice [89]. Clinical studies have established a positive correlation between hyperuricemia and markers of metabolic syndrome in young adults and in patients with asymptomatic hyperuricemia [90,91]. Importantly, treatment with allopurinol reduced these markers in patients with hyperuricemia. Increased XOR activity and hyperuricemia can promote hypertension through various mechanisms described above such as activating inflammation, decreasing endothelial NO availability, mediating endothelial dysfunction, activating the RAAS, and vascular remodeling [92]. These reports support that the mechanisms mediated by XOR activity are also involved in the development of diabetes and hypertension, which are the leading risk factors for CKD.

The apical surface of the endothelial cells is covered by the negatively charged endothelial glycocalyx, a hydrated gel-like structure consisting of glycoproteins and proteoglycans covalently linked to the glycosaminoglycans (GAG), heparan sulfate, chondroitin sulfate, and hyaluronic acid [93]. The release of XOR into circulation from organs such as the liver promotes the accumulation of XO in peripheral endothelia by binding to GAGs at the plasma membrane [8]. The XO binding to GAGs in human endothelial cells has been demonstrated with a *K_d_* of 6 nM, which is extremely favorable and underscores the potential for consequences to high XO accumulation and activity [8]. Once bound to endothelial GAGs, XO can mediate endothelial cell damage by inhibiting NO production through uncoupling eNOS and impact the vascular tone critical for the development of hypertension [38,72,94,95,96]. This reaction can also result in the production of peroxynitrite (ONOO-), an extremely potent reactive species that can do extensive damage, by nitrating protein residues on tyrosine leading to functional impairment of cells or cell death [97]. The glomerular endothelial glycocalyx rich in GAGs has been suggested to serve as a vulnerable site for XOR accumulation in diabetic mice [Figure 2, above], and shedding of the glycocalyx in the glomerulus would result in XO release, promoting endothelial dysfunction and glomerular injury and proteinuria [7,98].

Indeed, type 2 diabetes (T2D) and XOR activity have been suggested to play a role in diabetic kidney disease (DKD) pathogenesis [39,90,99,100]. Oxidative distress induced by XOR was also shown to be critical in the development of DKD, through ROS-mediated cellular damage and activation of cell death pathways [5,44]. Importantly, in humans, XO and UA were reported to be independent predictors of albuminuria, where an elevation in 1 µmol/L UA or 1U/L of XO increases albuminuria by 1.5% [101]. Mouse and rat studies have explored the effects of XOR inhibition in T2D, and showed anti-inflammatory effects, reduced distress, and reduced UA levels [102]. However, recent metanalyses in humans found that administration of allopurinol in DKD did not significantly improve renal function nor albuminuria, yet another metanalysis showed a significant reduction in UA levels with the use of allopurinol and preserved renal function in addition to reducing proteinuria [103,104]. Also, outcomes from clinical studies concluded that 3 years of sustained reductions of blood levels of UA with allopurinol did not benefit diabetic patients with mild to moderate kidney disease [105,106]. The studies’ conclusions have been disputed by the community because both studies included large numbers of patients with normal serum UA levels, and therefore they did not test whether treating hyperuricemia was beneficial, but rather tested whether lowering UA would be beneficial, and it is well recognized that normal UA levels do not increase the risk for CKD [107]. It should be noted that allopurinol while lowering UA, can cause excess ROS production through its recognized action of self-oxidation to form oxypurinol, ultimately leading to electron transfer to FAD, resulting in the reduction of O_2_ and more ROS formation [108,109,110]. Other reports have shown that allopurinol has off-target consequences, inhibiting other enzymes in the purine and pyrimidine metabolic pathways, which can also lead to kidney damage [111]. Non-purine XOR inhibitors have been shown to inhibit XOR-derived ROS damage and renal injury with greater selectivity than purine mimetics [112,113]. Febuxostat is one such drug, and although its use has had controversial outcomes, meta-analyses show the benefit of febuxostat in CKD and its superiority over allopurinol [113,114,115,116,117,118]. Randomized controlled trials involving 65 patients with hyperuricemia concomitant with diabetic nephropathy demonstrated that topiroxostat, another non-purine XOR inhibitor decreased serum uric acid and prevented a decline in renal function, without improving albuminuria in patients with diabetes [119], while in some other randomized controlled trials, topiroxostat displayed antialbuminuric effects [120,121]. The differences in observed effects of XOR inhibition may be attributed to the sample size of these clinical studies, duration of treatment, stage of patients’ CKD, levels of uric acid, or the drug effects as described above. In summary, although mixed results have been observed from pre-clinical to clinical studies with XOR inhibitors in diabetic and non-diabetic CKD summarized in Table 1 below, there is substantial evidence for the role of XOR in CKD pathophysiology and the beneficial effects for XOR inhibition in CKD.

Therapeutic targeting of XOR activity may, in fact, benefit a subgroup of patients [107,133]. This is supported by reports demonstrating genetic variations in XOR are significantly associated with CKD in a cohort of hypertensives [134]. While XOR activity can play a consequential role in the development of kidney diseases, unrelated renal insults, such as polycystic kidney disease (PKD), an inherited disorder, have also been correlated to changes in XOR activity [135]. PKD is a common genetic disease present in 1:400 people and is driven by either autosomal dominant or recessive genes [136,137]. Described by the development of cysts in the kidney, PKD exhibits oxidative distress, insults to insulin signaling, hyperuricemia, and endothelial dysfunction, which are linked to XOR activity in PKD [135,138,139]. In autosomal dominant PKD (ADPKD), hyperuricemia develops as renal function declines, underscoring the positive correlation with XOR activity as the disease progresses [140]. Interestingly, hyperuricemia in PKD has also been found to be a risk factor for nephromegaly and an earlier onset of hypertension [43]. Targeting XOR with febuxostat in PKD was shown to be beneficial by increasing patients’ eGFR, which supports the pathologic role of XOR activity in this condition [141]. Studies focused on the role of XOR activity in the development and progression of PKD have only recently begun, and an understanding of the mechanisms involved is just emerging. Future studies on how XOR and its products contribute to the onset and progression of PKD are warranted.

## 4. XOR Activity & Diet

While disease settings such as diabetes, hypertension, and PKD can influence XOR activity, environmental, and dietary factors may also mediate XOR activity [142,143]. XOR activity in driving hyperuricemia is greatly impacted by dietary patterns, such as purine-rich and western diets (WDs) [6,9,143]. Purine-rich diets consist of frequent intake of shellfish, organ meats, legumes, and alcohol [142,144,145]. Ingestion of purine-rich foods supplies the highly active purine salvage pathway, which is vital in producing nucleic acids for bioenergetic regulation, cellular replication, and sustaining transcriptional and translational activities through the generation of mRNA and tRNAs, and this has been elegantly demonstrated in model organisms such as *E. coli* and *S. cerevisiae* [146]. While de novo purine synthesis can occur, it is energetically expensive, whereas dietary sources of purines are abundant and can be modified with much less energy expenditure in the form of ATP [147]. Thus, dietary purine contents have been suggested to regulate UA production by XOR and form the basis for nutritional interventions in hyperuricemic diseases—predominantly gout [144,148]. While purine-rich diets directly contribute to XOR activity by means of supplying upstream substrate(s), WDs, notably high in saturated fats, fructose, and cholesterol, have also been associated with increased XOR expression and activity with hyperuricemia, gout, and metabolic diseases [149,150]. WDs have been implicated as major drivers of insulin resistance [151]. WDs, which often have subjects consuming nutrient-dense, ultra-processed foods more frequently, promote oxidative stress and metabolic disease [151,152,153]. Nitrites, a major mineral involved in food processing in WDs, directly impact XOR activity to reduce nitrites to NO [154,155]. This mechanism may act as a compensatory role for NO production by eNOS when XOR mediates the uncoupling of eNOS [156]. However, more investigation into the mechanism of WDs effect on XOR activity and its impact on health is needed.

### 4.1. Diet-Induced XOR Activity in CVD and Fatty Liver

Chronic WD exposure is a recognized and costly risk factor for cardiovascular disease (CVD), supported by numerous studies demonstrating that WD can increase cardiac distress, inflammation, and cell death [157,158,159,160]. WD has been shown to also alter cardiac contractility in rats as a result of dysregulated fatty acid metabolism [161]. Moreover, advanced glycation end products, which are markers for organ damage and inflammation, were found to accumulate in mice fed a WD, suggesting activation of receptors for advanced glycation end products, is consequential in WD-induced CVD [158]. In an ApoE knockout mouse model of atherosclerosis, dietary tungsten inhibited XO, and mitigated the development of atherosclerosis from WD, suggesting that dietary tungsten, could have a positive impact on WD-induced XOR activity [Table 2, below] [162]. Risks for cardiac insults in humans are increased during hyperuricemia, having shown that diets high in sodium, typical in some WDs, are associated with increased XO activity and left ventricular hypertrophy in resistant hypertension [15]. WDs directly impact hepatic health and the susceptibility to developing the metabolic-associated fatty liver disease (MAFLD) [163,164]. While predominantly produced and released by the liver, hepatic XOR plays a focal role in the progression of MAFLD [6,165]. WDs promote insulin resistance, which in turn drives the pathogenesis of MAFL and metabolic-associated steatohepatitis (MASH). In mice challenged with WD, XOR inhibition was able to attenuate insulin resistance and mitigate the progression of MASH [6,166]. Interestingly, a recent study revealed that endothelial-sourced released hypoxanthine was increased in vitro, suggesting that activated endothelia directly promotes XOR activity by providing metabolic substrates for XOR [Figure 2, above] [167]. Kawachi et al. treated HUVECs primed by human liver S9—a fraction of microsomal and cytosolic enzymes from the liver, which are rich with XOR—with the selective XOR inhibitor, topiroxostat, and found that hypoxanthine was still highly produced compared to attenuated levels of xanthine and UA [167]. This further highlights the negative impact of XOR on endothelial function as well as the complex relationship between XOR regulation, endothelial activation, and dysfunction, and its contribution to CVD and MAFLD pathogeneses [168].

### 4.2. Diet, XOR Activity in CKD

High fructose intake has been demonstrated to impact renal health and function in humans, increasing the risk for hyperuricemia and kidney stones [169]. Recent evidence in mice revealed that WD-induced MASH promoted the development of CKD, which can be mitigated by healthy liver transplantation, suggesting that a hepatorenal relationship is responsible for CKD progression in this setting [170]. Indeed, there is mounting research suggesting a strong causal role for fructose consumption on UA production; including a study in mice showing that UA can stimulate hepatic fructokinase in a seemingly vicious positive feedback loop promoting metabolic disease [4,171]. Likewise, dietary fructose in mice has been shown to induce de novo purine anabolism, which can strain downstream purine catabolism by XOR [143]. Indeed, recent studies employing transcriptomics and metabolomics in livers from mice fed high fructose, underscore the causal relationship between fructose feeding and XOR activity, demonstrating significantly increased gene expression of purine metabolic enzymes, especially XOR, as well as elevated hypoxanthine, adenine, adenosine, AMP, and guanine metabolite levels [143]. Proximal tubules are responsible for fructose reabsorption for renal gluconeogenesis and are susceptible to high fructose diets resulting in tubulointerstitial injury, supporting the notion that high fructose feeding is associated with poor outcomes, promoting CKD pathogenesis [172,173,174]. In vitro studies have found that fructose administered to HK-2 cells promotes fructokinase activity and subsequently the production of proinflammatory factors [175]. In light of this evidence, strategies to mitigate fructose-induced renal damage and fibrosis are becoming increasingly important. Recent advances in glucagon-like peptide-1 (GLP-1) receptor agonists, such as the use of dulaglutide, have shown promising results in reducing renal fibrosis, in albino rats chronically fed high fructose [176]. Indeed, a study in Taiwan found that GLP-1 receptor agonists provide renal benefits in T2D patients in contrast to long-acting insulins [177]. Altogether, dietary patterns can play a role in mediating XOR activity and future research exploring nutritional interventions could be beneficial in managing or even mitigating chronic diseases like CKD [178].

**Table 2:** Factors regulating XOR and outcomes.

**Factor**	**Impact on XOR** **Expression/Activity**	**Biological Effect**	**Effect Size**	**Reference**
Purine-rich Diet	Increase	Greater UA; elevated UA precursors promote purine catabolic activity of XOR	Odds ratio: 1.024 for animal-derived foods	[145]
Western Diet	Increase	Greater UA	Odds ratio: 2.15 for animal-derived and fried foods	[179]
Tungsten	Reduce	Reduced ROS; Prevents Molybdenum incorporation at the MoCo site thereby reducing XO-derived ROS production.	N/A	[180,181]
Heat	Increase	Renal function (measured in eGFR) declined with rising UA. Electrolyte hydration by sugarcane workers appeared preventative to the decline	adj β = −10.4 and 8.1, respectively	[182]
Xanthinuria	Non-functional	Reduced UA; elevated purines and formation of xanthine stones	1:69,000 people (combined incidence of hereditary types I and II)	[183]
*XDH* rs207454	Reduce	Improved survival for ‘C/C’ carriers facing gastric cancer compared to ‘A/A’ or ‘A/C’ carriers.	Hazard Ratio: 1.53 for A/A and A/C carriers	[184]
*XDH* rs185925	Increase	Higher XOR expression was associated with acute respiratory distress syndrome in septic African American patients	Odds ratio: 1.464 for C/T carriers	[23]
*XDH* rs1884725	Increase	Increased serum creatinine (a predictor for renal dysfunction) in septic African American patients.	β = 0.504	[23]
*XDH* rs4952085	Increase	Increased serum creatinine (a predictor for renal dysfunction) in septic African American patients.	β = 0.493	[23]

## 5. XOR Activity and the Environment

### 5.1. XOR and Heavy Metal Exposures

Susceptibility to environmental impacts beyond diets on XOR activity and its products has also been explored. Studies have found that XORs are susceptible to dysregulation as a result of toxic metal intake, excessive heat exposure, and infectious diseases [23,80,185,186,187]. Heavy metal (e.g., lead, copper, tungsten, etc.) exposure can come from a variety of sources and has been positively associated, particularly lead, with the development of hyperuricemia in gout patients [188]. Interestingly, copper has also been suggested to play a role in dysregulating XOR activity and may have a greater impact on people compared to lead, due to its wide dietary availability [185]. On the other hand, dietary tungsten has been used to inhibit XOR activity and does so by displacing molybdenum due to similar bonding properties in the same periodic group [162,189]. More research focused on the impact of heavy metals on XOR activity could help better our understanding the impact of heavy metal exposure on human health and toxicity.

### 5.2. XOR and CKDu

With climate change causing increased temperatures, more and more people are being chronically exposed to extreme heat, which is associated with an increased risk of CKD [190,191]. A recent study in mice employed heat stress to significantly induce renal injury and found that treatment of allopurinol mitigated the fibrotic and inflammatory effects that were observed in heat-stressed mice, suggesting that XOR activity is triggered in chronic heat stress and may mediate kidney injury in this context [186].

Over the past decades, there has been an increase in the prevalence of CKD of unknown etiology (CKDu). First, reported amongst young rural farming communities in El Salvador, CKDu has since been reported in regions of the world such as India and Sri Lanka [192,193,194,195]. CKDu has also been reported among miners, and construction and transportation workers living in similar hotter and lower altitudes areas of the U.S. Pacific coast [196]. CKDu is typically asymptomatic, however, individuals exhibit increased serum creatinine with little or no proteinuria [192,197]. Additionally, kidney biopsies of affected individuals reveal tubulointerstitial disease which typically progresses to renal failure [198]. Since the first observation of CKDu was made, various pathophysiologic mechanisms for development have been proposed [193]. Based on studies over the years, Dr. Johnson and colleagues propose that the ‘chronic recurrent dehydration’ hypothesis is most plausible [182,196]. This hypothesis proposes that high levels of physical activity, with insufficient hydration in hot environments, lead to dehydration, increased serum osmolarity, and subclinical-grade muscle injury [Table 2, above]. Subclinical-grade muscle injury and rhabdomyolysis lead to the release of purines from damaged muscles, which eventually results in increased XOR and production of UA from these purine substrates. This, along with volume depletion due to dehydration leads to hyperuricemia. UA in the setting of hyperuricemia can cause urate crystal deposition in renal tubules and contribute to the acidification of urine, which together initiate events that lead to tubular injury [64,199]. Other reports support the common occurrence of hyperuricemia in individuals with CKDu [200,201]. In the quest to understand the cause of CKDu, other investigators undertook multi-omics approach by exposing Zebrafish to water from a CKDu-prevalent region of Sri Lanka [202]. The researchers identified necroptosis and purine metabolism pathways to be dysregulated in CKDu [202]. Although heat exposure, possible concomitant particulate matter, and toxicants exposure in these environments may play a role in promoting CKDu in those at risk, the role of XOR and UA in the pathogenesis of the disease may be important [203,204,205].

## 6. Genetic Regulation of XOR

While components of diet and the environment have some effect on XOR expression and activity, a recent metanalysis from ARIC, CARDIA, CHS, FHS, and NHANES III studies in the U.S., using 16,760 patients (8414 men and 8346) of European descent demonstrated that genetic factors, specifically single nucleotide polymorphisms (SNPs) in 30 different genes, play a systemic and consequential role in mediating serum UA [148]. The genetic loss of functional human *XDH* results in renal decline, which is reflected in a hereditary xanthinuria model of *XDH* loss in rats [Table 2, above] [42,45]. There also exists genetic polymorphisms in *XDH* (rs207454), which influence gastric cancer survival in Chinese patients and breast cancer survival among a Spanish cohort through increased *XOR* expression [184,206,207]. However, in certain scenarios, the regulation of the *XOR* gene could potentially contribute to disease progression in various settings. The human XOR is transcriptionally regulated by the presence of E-box and TATA-like elements in the promoter region (−258 to −1) of *XDH* [208]. In a study evaluating risk for sepsis and associated organ failures, six intronic variants enriched with regulatory elements for *XOR* were associated with risk of sepsis among African Americans (rs206805, rs513311, rs185925, rs561525, rs2163059, rs13387204) [Table 2, above]. Interestingly, the rs185925 variant was identified to influence XOR activity, and two common SNPs (rs1884725 and rs4952085) were in tight linkage disequilibrium which provided strong evidence for association with increased levels of serum creatinine and risk of renal dysfunction [23,184]. Conversely, the same study found that the missense variant rs17011368 was associated with enhanced mortality in septic European Americans with acute respiratory distress syndrome compared to African Americans [23]. Thus, variations in *XDH* regulation are associated with risk for several negative outcomes, and differences in ethnic risk profiles may begin to explain some of the health disparity (i.e., higher morbidity and mortality) in African American patients.

In line with the epidemiologic evidence from human studies, promoter polymorphisms of *XOR* have been associated with increased activity and gene expression, and increased XOR in circulation is strongly associated with ESRD [209,210]. In an unbiased integrative systems-genetics study in mice, our group identified the presence of a variation in the C/ebpβ binding element of *XDH* in DBA/2J mice with high risk for kidney injury phenotype, compared to resistant phenotype exhibited by C57BL/6J mice in the setting of diabetes [5,73]. By substituting the low-risk with high-risk variants from the DBA/2J strain using CRISPR Cas9, Wang et al. were able to generate a kidney injury susceptible C57BL/6J mouse model; B6-*Xor^em1^*, where B6-*Xor^em1^* mice exhibited significantly higher levels of XOR activity with superimposed diabetes, high-fat, diet-induced metabolic syndrome and with aging [5]. Mechanistically, the study confirmed that these variants could influence the binding of transcription factor C/ebpβ. Although C/ebpβ is normally expressed at low levels in kidneys, this study detected higher C/ebpβ expression in glomeruli of diabetic DBA/2J and B6-*Xor^em1^* mice but not in diabetic C57BL/6J. Primary GECs from B6-*Xor^em1^* mice showed nuclear translocation of C/ebpβ with high glucose or oxidized LDL, while primary podocytes from diabetic C57BL/6J did not. RNA interference for *C/ebpβ* prevented the high glucose-induced *XOR* expression and ROS production, confirming its role in modulating XOR activity in a high glucose milieu. Furthermore, the study demonstrated that high-risk strains (DBA/2J and B6-*Xor^em1^*) had increased XOR activity with concomitant oxidative distress in endothelial cells, glomerular injury, and proteinuria, which could be rescued by febuxostat treatment. The data underscores the critical role of *Xor* regulation in influencing kidney injury in diabetes, which is also relevant to other diabetic complications and aging [211,212,213]. Importantly, *XOR* promoter orthologues were identified in humans having diabetic complications including kidney disease in the UK BioBank and in the Mount Sinai Bio*Me* Biobank [5]. Identifying individuals at risk for developing DKD or other diabetic complications is crucial for early intervention, prevention of further complications, and improving overall health outcomes and quality of life for those individuals. From a mechanistic standpoint, this work advocated for a precision-medicine approach, in which genetic risk variants in the *XOR* promoter that result in increased activity could potentially guide treatment decisions. Future research will evaluate whether *XOR* promoter variants could be potential genetic biomarker predictors of progressive disease, aiding in the identification and treatment of those at risk.

## 7. Novel Therapeutic Approaches Targeting XOR

The use of current XOR inhibitors is associated with serious side effects that limit their use in patients. For instance, purine derivatives such as allopurinol have been associated with gastrointestinal distress, renal toxicity, rash, and eosinophilia [111,214]. Non-purine structured inhibitor febuxostat is associated with an increased risk of cardiovascular mortality and in 2017, it received a black box warning from the FDA [215]. Considering the established involvement of XOR in several diseases including kidney disease, there have been global efforts geared towards the development of effective pharmacological inhibitors of XOR that do not cause noteworthy adverse reactions. Optimism remains around tackling the aberrant purine metabolism in the development of ADPKD and DKD; XRx-008 and XRx-225, novel formulations of oxypurinol that have been designed to slow or reverse disease progression are currently in Phase II and Phase I clinical trials, respectively. Recently, candidate non-purine compounds, namely ALS-1, -8, -15, and -28, have been shown to effectively inhibit XO in a reversible manner [216]. These compounds may offer a safer therapeutic option for disorders that are characterized by a dysregulation in XOR activity. Other approaches aim to combine the inhibition of both XOR and URAT1 in order to reduce uric acid and avoid the issue of insufficient potency by single-target drugs [217]. Progress in this space over the last 20 years has been captured in a comprehensive review elsewhere, which highlights the use of novel drug design approaches such as fragment-based drug design or molecular hybridization, or the combination of both in designing a new class of XOR inhibitors that could potentially effectively eliminate side effects while achieving the desired potency and efficacy [218]. Interestingly, the development of new inhibitor candidates for XOR so far is largely based on derivatives inspired by existing drugs, however, this approach has resulted in limited success. Therefore, new approaches such as the use of exogenous UOX (i.e., enzyme replacement therapy) to lower UA levels could be further explored in the setting of gouty nephropathy [219]. Despite the challenges that are being encountered by the currently approved inhibitors of XOR, pharmacological targeting of XOR still holds enormous therapeutic potential, especially for chronic diseases that are associated with increased XOR activity in non-diabetic and diabetic kidney diseases. Exciting and new emerging trends in this research are becoming evident, as scientists are exploring natural compounds for XOR inhibition with pleiotropic effects, there are more developments for new tools for identifying drug candidates that target optimal efficacy and safety profiles, as well as efforts to identify the patients who may be most responsive.

## 8. Conclusions

**Figure 3:**
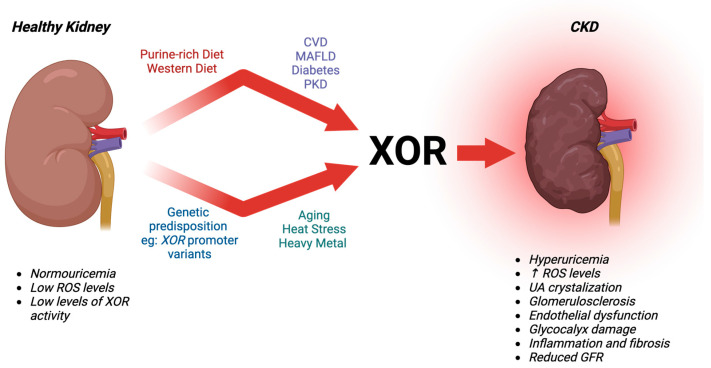
**XOR’s Influence on Driving CKD.** XOR directly contributes to CKD by increasing oxidative distress and the production of UA. Subsequently, XOR contributes to glomerular endothelial cell dysfunction, inflammation, and fibrosis. Genetic predisposition, environmental factors such as diet and heat, and aging can also contribute to total circulating XOR and renal pathogeneses. CVD, cardiovascular disease; GFR, glomerular filtration rate; MAFLD, metabolic-associated fatty liver disease; PKD, polycystic kidney disease; ROS, reactive oxygen species; UA, uric acid.

In conclusion, XOR activity is complex in health and dysregulation can be induced by a myriad of environmental factors, such as diet, heat, toxic exposure(s), or diseases, or aging. It is important to note that there are sex differences in the expression of XOR in humans and animal models [220,221,222]. The sex-based differences can influence XOR expression and activity potentially leading to higher susceptibility to chronic diseases [221,223]. Considering XOR in CKD, there are promising results from current therapeutics at alleviating some key factors involved in CKD pathogenesis; however, to avoid off-target effects novel therapeutics should be more thoroughly investigated for clinical potential. Exciting new evidence on the genetics of *XOR* regulation on susceptibility to diseases, especially wherever oxidative damage is involved, is of particular importance. Genetic polymorphisms of *XOR* could serve as valuable biomarkers of susceptibility, aiding in the identification of patient sub-groups that would benefit from targeted therapies. Understanding the interaction of *XOR* and various influencing factors such as diseases, aging, diet, toxicant exposures, and climate change, all potentially leading to CKD [Figure 3, below], will assist in accurately diagnosing individuals at risk and providing them with more precise and effective treatments.

## Figures and Tables

**Table 1:** Selected preclinical and clinical studies of XOR inhibitors in CKD.

**Pre-Clinical Studies**				
**Disease**	**Model(s)**	**Study Design**	**Findings**	**Reference**
DKD	Akita mouse (genetic model of type 1 diabetes mellitus)	Akita mice were treated with topiroxostat, a non-purine inhibitor of XOR for 4 weeks, and markers of XOR activity and diabetes-induced glomerular injury were measured.	Compared to non-diabetic C57BL/6J controls, vehicle-treated Akita mice developed progressive diabetic glomerular injury, which was associated with increased XOR activity and oxidative stress. Pharmacological inhibition of XOR activity with topiroxostat markedly decreased renal ROS and progressive DKD in Akita mice.	[7]
DKD	*db*/*db* mouse (model of obesity and type 2 diabetes mellitus)	*db*/*db* mice were treated with various doses of XOR inhibitors (topiroxostat and febuxostat) for 4 weeks, and markers of systemic and renal XOR activity and kidney injury were assessed.	Compared to control *db*/*m* littermates, control *db*/*db* mice exhibited increased systemic and intra-renal XOR activity, and this was associated with proteinuria. Treatment with topiroxostat significantly decreased XOR activity and oxidative stress in *db*/*db* mice. More importantly, topiroxostat rescued proteinuria in *db*/*db* mice in a dose-dependent fashion. While febuxostat significantly inhibited XOR activity in *db*/*db* mice, it did not significantly decrease proteinuria.	[122]
DKD	Streptozotocin-induced diabetic Sprague Dawley rat (chemically induced model of type 1 diabetes mellitus)	Diabetic Sprague Dawley rats were treated with febuxostat for 7 weeks, and markers of XOR activity and renal oxidative stress, renal macrophage infiltration, and proteinuria were measured.	In comparison to non-diabetic controls, diabetic Sprague Dawley rats exhibited increased XOR activity, renal inflammation, and renal injury, which were attenuated by treatment with febuxostat.	[113]
DKD	Akita, HFD-fed mice, STZ-induced diabetic B6-TG mice, and recombinant inbred BXD mice	This study employed multiomic and pharmacological approaches using the XOR inhibitors, allopurinol and febuxostat, to study the role of a *XOR* risk variant in the development of DKD	This study discovered that this identified variant is in the *XOR* promoter region and is a transcription factor binding site for C/EBPβ. Importantly, findings from this study demonstrated that the identified variants confer susceptibility to DKD.	[5]
CKD Non-DKD	5/6th nephrectomy Wistar rats with oxonic acid-induced hyperuricemia	5/6th nephrectomized Wistar rats were made hyperuricemic with oxonic acid and treated with febuxostat for 4 weeks. Plasma uric acid, renal hemodynamics, proteinuria, and histopathologic evaluation were carried out in response to treatment with febuxostat.	5/6th nephrectomized rats treated with oxonic acid displayed hyperuricemia, impaired renal hemodynamics and proteinuria versus vehicle-treated 5/6 nephrectomy rats. The administration of febuxostat decreased hyperuricemia, improved renal function, and ameliorated proteinuria in 5/6th nephrectomized rats that received oxonic acid.	[123]
Obstructive nephropathy	Rat model of unilateral urethral obstructive (UUO) nephropathy	Sprague Dawley rats were pretreated with febuxostat before UUO surgery and then daily after surgery for up to 14 days. Rats were sacrificed on days 1, 4, and 14 after surgery. Markers of intrarenal and systemic XOR activity, oxidative stress, renal inflammation, and tubulointerstitial fibrosis were assessed in response to treatment with febuxostat.	Vehicle-treated UUO rats displayed increased XOR activity, renal inflammation, and tubulointerstitial fibrosis in comparison to sham rats. Pharmacological blockade of XOR with febuxostat rescued renal oxidative stress and inflammation and decreased tubulointerstitial fibrosis in UUO rats.	[124]
Hypertension-induced CKD	Dahl salt-sensitive rat (genetic model of salt-sensitive hypertension and renal injury)	Dahl salt-sensitive (SS) rats fed a high salt (8% NaCl) diet and treated with febuxostat daily for 4 or 8 weeks. XOR expression and XOR activity, oxidative stress, and progressive renal disease were measured in febuxostat-treated SS rats and their controls.	Compared to normal salt (0.6% NaCl) diet-fed SS rats, high salt-diet-fed SS rats exhibited increased renal XOR expression and XOR activity, which was associated with oxidative stress and the progression of renal disease in SS rats. Treatment with febuxostat decreased XOR expression and activity and attenuated the progression of renal disease in high salt diet-fed SS rats.	[125]
IgA Nephropathy	gddY mouse (rodent model of IgA nephropathy)	gddY mice either served as controls, or were treated with febuxostat for 9 weeks, during which XOR activity, inflammatory signaling, and the progression of renal disease were investigated.	Control gddY mice demonstrated increased XOR activity, renal inflammation and fibrosis and renal disease versus BALB/c controls. XOR blockade with febuxostat was associated with decreased renal inflammation and renal disease in gddY mice.	[126]
Hyperuricemic Nephropathy	Adenine + potassium oxonate-induced hyperuricemic Sprague Dawley rats.	Control or febuxostat-treated hyperuricemic rats were treated for 5 weeks, and markers of XOR activity, endoplasmic reticulum stress, apoptosis, and renal disease were assessed.	Treatment with febuxostat decreased XOR activity and renal urate deposition versus controls. Importantly, treatment with febuxostat ameliorated renal disease in hyperuricemic rats.	[127]
**Clinical Studies**				
**Disease**	**Study Population**	**Study Design**	**Outcomes**	**Reference**
DKD	Patients with diabetes, concomitant with hyperuricemia	Randomized, double-blinded, placebo-controlled, and parallel study of patients with DKD and hyperuricemia. Recruited patients received either placebo or topiroxostat for 28 weeks. uACR was primary endpoint, while eGFR and serum uric acid levels were secondary endpoints.	Topiroxostat decreased serum uric acid, while preventing renal function decline in the study population. However, topiroxostat did not have a significant effect on uACR in comparison to placebo.	[119]
DKD	DKD patients without gout	Cross sectional study of type 2 diabetes patients without a history of gout or allopurinol use. Serum creatinine and uric acid, HbA_1C,_ and 24-hr urine protein excretion were measured.	Serum uric acid showed a positive correlation with proteinuria in diabetic patients.	[128]
DKD	Patients with diabetes and asymptomatic hyperuricemia	Randomized, parallel-controlled trial that investigated the effects of allopurinol on renal function in diabetic patients and hyperuricemia.	Effective control of serum uric acid with allopurinol was associated with decreased albumin excretion and the prevention on renal function decline in the study population.	[129]
Hyperuricemic CKD	Hyperuricemic patients with stage 3 CKD	Randomized, open label, parallel-group trial to investigate the renoprotective effects of febuxostat on hyperuricemic patients with stage 3 CKD.	Treatment with febuxostat decreased serum uric acid and urinary levels of albumin and β2-macroglobulin in hyperuricemic CKD patients versus the control group. However, febuxostat did not influence markers of renal function (serum creatinine and eGFR).	[130]
Hyperuricemic CKD	Hyperuricemic stage 3 CKD patients, with or without gout	This was a 22 week double-blind, multicentric, randomized trials that studied the safety and efficacy of topiroxostat in hyperuricemic patients with stage 3 CKD.	Topiroxostat decreased serum urate and uACR in hyperuricemic stage 3 CKD patients versus placebo group. The observed effect of topiroxostat on renal function (i.e., eGFR) was not statistically significant. Adverse events were generally comparable between topiroxostat and placebo groups.	[131]
Hyperuricemic CKD	Hyperuricemic patients with stage 3–4 CKD	Randomized controlled trial that examined the efficacy of febuxostat on markers of endothelial dysfunction and renal function in CKD patients with asymptomatic hyperuricemia.	Compared to the control group, febuxostat reduced serum uric acid and preserved renal function. However, febuxostat did not significantly decrease albuminuria or markers of endothelial dysfunction versus controls.	[132]
